# Paternal and maternal psychological distress and adolescent health risk behaviors: The role of sensitive periods

**DOI:** 10.1002/jad.12385

**Published:** 2024-07-28

**Authors:** Maria Sifaki, Eirini Flouri, Emily Midouhas

**Affiliations:** ^1^ Research Department of Epidemiology and Public Health UCL Institute of Epidemiology and Health Care London UK; ^2^ Department on Psychology and Human Development UCL Institute of Education London UK

**Keywords:** adolescent health risk behaviors, maternal psychological distress, Millennium Cohort Study, paternal psychological distress

## Abstract

**Introduction:**

Adolescent health risk behaviors are linked to poor physical and mental health outcomes. While past research shows that maternal psychological distress predicts those behaviors, we know less about the role of paternal psychological distress and the role of sensitive periods.

**Methods:**

Using 11,128 data from families (50.5% female children) from the UK's Millennium Cohort Study, we examined the role of timing of exposure to paternal and maternal psychological distress in engagement in health risk behaviors (smoking, alcohol use, binge drinking, and sexual activity) at age 14. Paternal and maternal psychological distress, measured with the Kessler‐6 scale, were assessed at child ages 3, 7, and 11. We performed path analysis, adjusting for key covariates, modeling maternal distress parallel to paternal, and allowing for autoregressive paths.

**Results:**

Paternal distress experienced at age 11 predicted a higher likelihood of smoking at age 14. Maternal distress at age 7 also predicted a higher likelihood of smoking, alcohol use, and binge drinking, but only for boys. Moreover, maternal distress at age 3 was associated with a lower risk for alcohol use. Effects were not replicated in the sensitivity analysis we performed, including only families with resident biological fathers across the study period. Instead, maternal and paternal distress at age 11 raised girls' risk for binge drinking and sexual activity, respectively.

**Conclusions:**

Parental distress in early childhood does not predict adolescent health risk behaviors. In late childhood, however, both paternal and maternal distress seem to influence the likelihood of engagement in such behaviors.

## 1 INTRODUCTION

Adolescent health risk behaviors such as smoking, drinking, and sexual activity, are major public health concerns, as they are linked to a range of adverse physical and mental health outcomes both in the short and in the long term (Boden et al., 2020, 2010; Enstad et al., 2019; Epstein et al., 2014; Levola et al., 2020). The timing of onset of these behaviors also seems critical. For example, having initiated those behaviors before the age of 14 is associated with severe forms of substance abuse in later life (Hingson et al., 2006; Silva et al., 2023). Pinpointing the risk factors responsible for the emergence of such behaviors is an essential step toward prevention.

Previous studies suggest that maternal psychological distress may play a role in these health‐risk behaviors in adolescence (Ali et al., 2016; Flouri & Ioakeimidi, 2018; Herman‐Stahl et al., 2017, 2008; Lamis et al., 2012; Sang et al., 2016; Wickham et al., 2015). These studies indicate that the timing of distress symptoms matters, with middle and late childhood being the most impactful. Specifically, Flouri and Ioakeimidi (2018) found that sons (but not daughters) of mothers with elevated distress levels in late childhood (around 11 years old) were more likely to engage in antisocial behavior, including alcohol use. There were no effects for offspring whose mothers experienced distress at younger ages. Furthermore, Wickham et al. (2015) identified maternal depression trajectories across child ages 2–14 and assessed how these relate to youth risk engagement at age 16. Results showed that adolescents exposed to high maternal distress in middle childhood (around 8 years old) initiated smoking and drinking earlier, compared to those exposed to consistently low maternal distress symptoms (the reference group).

Accumulating evidence suggests that paternal distress may also affect adolescent outcomes. Prior studies using data from the Millennium Cohort Study (MCS, the same data set the current study uses) have linked paternal distress to increased internalizing and externalizing difficulties, particularly for boys (Flouri et al., 2019; Sifaki et al., 2021). These associations were independent of the influence of maternal distress, which further emphasizes the important role of fathers. In addition, research studies indicate that the paths between paternal distress and adolescent difficulties may be transactional (Sifaki et al., 2021; Speyer et al., 2022).

However, we know relatively little about the influence of paternal distress in the development of adolescent health risk behaviors (Ali et al., 2016; Essau & de la Torre‐Luque, 2021; Herman‐Stahl et al., 2008). For example, analyzing data from the National Surveys on Drug Use and Health (NSDUH), Herman‐Stahl et al. (2008) explored the impact of severe paternal and maternal psychological distress on adolescent binge drinking and illicit drug use. Having a mother with severe psychological distress heightened the risk of engagement in both behaviors. However, severe psychological distress predicted lower risks for binge drinking, but only for black families. According to the study authors this may be that, within this ethnic group, family members become very supportive of each other's struggles, and that may lead adolescents whose fathers face severe distress to behave responsibly and avoid risky behaviors. Using the same data source, Ali et al. (2016) examined how parental comorbid mental health and substance abuse disorder was linked to adolescent substance abuse (alcohol or drugs) disorder. Findings showed that a significant path existed only for mothers, not fathers. Finally, Essau and de la Torre‐Luque (2021), using data from the National Comorbidity Survey Replication Adolescent Supplement (NCS‐A), identified psychopathology and substance abuse trajectories for fathers and mothers. Two trajectories emerged for fathers: “low” (low psychopathology symptoms and substance use) and “high” (high psychopathology symptoms and drug use). Adolescents whose fathers belonged in the “high” trajectory faced increased risks for illicit drug abuse. These findings suggest that offspring of distressed parents are more likely to engage in substance misuse, possibly because they imitate their parents (distressed adults are more likely to misuse substances; Åhlin et al., 2015; Lawrence et al., 2009) or because their parents are unable to effectively monitor them (Botzet et al., 2019; Kelly et al., 2017). It is also possible that shared environmental adversities (e.g., living below the poverty line) independently affect both parental psychological distress and adolescent risky engagement (Gjerde et al., 2021).

Importantly, it may be critical to disassociate paternal from maternal distress in these links. For example, the direction of the effect of paternal distress on adolescent risky engagement is unclear, with one study supporting that it raises risks (Essau & de la Torre‐Luque, 2021), and another that it lowers them (Herman‐Stahl et al., 2008). Additionally, it is not known if there are key developmental stages during which the impact of paternal distress may be greater. Defining sensitive periods can help plan and implement interventions when they would be most beneficial. Another point to consider is that studies have not examined psychological distress per se in the general population as they focused either on “severe” forms of psychological distress or on co‐morbid distress and substance abuse*.* For example, in the studies by Ali et al. (2016) and Essau and de la Torre‐Luque (2021), discussed earlier, it is unclear if the effects observed are due to parental psychological distress or substance misuse. Finally, the role of maternal psychological distress was not consistently considered in the existing research, thus preventing clear conclusions about any unique paternal influences (Ali et al., 2016; Herman‐Stahl et al., 2008).

The present study aims to address these research gaps and expand the current knowledge of the effects of paternal psychological distress on adolescent health risk behaviors. Using longitudinal, general‐population data from the UK's MCS, it investigates how paternal psychological distress at ages 3, 7, and 11 (corresponding to early, middle, and late childhood, respectively) predicts adolescent health risk behaviors at age 14 while controlling for key confounders. Maternal psychological distress is modeled simultaneously and in the same way as paternal psychological distress, to factor out its impact and allow comparisons between the two. Considering that past research concluded that pathways from maternal psychological distress to adolescent risk behaviors may differ for boys and girls (Flouri & Ioakeimidi, 2018), effects are explored jointly as well as by sex.

## 2 METHODS

### 2.1 Participants

We used data from the UK's Millennium Cohort Study (MCS), an ongoing, population‐based cohort, including information on 19,243 UK families (19,517 children), who had a child born in 2000–2002 (https://www.cls.ioe.ac.uk/mcs). In the MCS, families were selected disproportionately, so that all UK minority groups in England, and disadvantaged wards across the United Kingdom, are sufficiently represented (Plewis, 2007). Data are available for 7 “sweeps” (i.e., data waves), which took place when children were aged around 9 months, and 3, 5, 7, 11, 14, and 17 years, respectively. In total, 18,552 families participated in sweep 1, 15,590 in sweep 2, 15,246 in sweep 3, 13,857 in sweep 4, 13,287 in sweep 5, 11,726 in sweep 6, and 10,625 in sweep 7. Ethical approval was gained from NHS Multi‐Center Ethics Committees. Parents gave informed consent, and children, at the ages of 11 and 14, gave informed assent and consent, respectively. For the current study, data from child ages 3, 7, 11, and 14 (sweeps 2, 4, 5, and 6, respectively) were used. The analytic sample, 11,128 cases, consisted of children and their families who met the following criteria:

1) Child was a singleton or first‐born twin or triplet.
2) Child had data on all measured health risk behaviors at age 14.
3) There was at least one paternal psychological distress score at child's ages 3, 7, or 11.
4) There was at least one maternal psychological distress score at child's ages 3, 7, or 11.

### 2.2 Measures

*Adolescent health risk behaviors* were assessed at age 14, through self‐report. Smoking was measured with a binary variable of ever smoked (including electronic cigarettes) or not. Alcohol use was assessed with a 3‐level categorical variable, asking whether the child: had never had an alcoholic drink, had had an alcoholic drink but had never engaged in binge drinking, and had engaged in binge drinking at least once. Binge drinking was defined as consuming five or more alcoholic drinks in one take. Last, sexual activity was measured with a dichotomous variable, demonstrating whether the child had ever engaged in any type of sexual activity or not (Gage & Patalay, 2021).

*Paternal* and *maternal psychological distress* were assessed with the 6‐item Kessler Psychological Distress scale (K‐6) at child ages 3, 7, and 11. K‐6 is a self‐administered measure of emotional state and overall distress, with good psychometric properties (Kessler et al., 2002). It evaluates depressive and anxiety symptoms experienced within the last month with the following 6 questions: “During the last 30 days, about how often did you feel: so depressed that nothing could cheer you up/hopeless/restless or fidgety/that everything was an effort/worthless/nervous?”. These items were rated on a five‐point scale, ranging from “none of the time” to “all the time”. Responses were added to create a final score, varying from 0 to 24, with higher values indicating more difficulties. Cronbach's alpha for fathers was .79, .83, .97, while for mothers it was .85, .87, .97, across child ages 3, 7, and 11, respectively.

*Key family‐level covariates* included factors related to both adolescent health risk behaviors and parental psychological distress: *family income, fathers'* and *mothers' alcohol use, fathers'* and *mothers' educational level*, and *fathers' biological status*. *Family income* (in our case, the poverty line) was assessed when children were aged 3, with a binary variable showing if the family income was above or below 60% United Kingdom's median household income (Kipping et al., 2015; Melotti et al., 2011). *Fathers'* and *mothers' alcohol use* were each measured at child age 11, with a 6‐item variable, reflecting how many alcoholic drinks caregivers consumed on an average day. Possible responses were “none,” “1–2,” “3–4,” “5–6,” “7–9,” and “10 or more” (Haugland et al., 2013; McCutcheon et al., 2018). *Fathers'* and *mothers' educational level* were each assessed using a binary variable, indicating whether the parent had or had not obtained a university degree by the time the child was aged 14 (Melotti et al., 2011). *Fathers' biological status* was also measured with a binary variable, which demonstrated if across ages 3–14 the child lived consistently with their biological father or not (McCutcheon et al., 2018).

The *child‐level variables* controlled for were *sex* (male or female), *ethnicity, internalizing difficulties, externalizing difficulties* as well as *pubertal status*. *Ethnicity* was assessed with a set of binary variables, specifying if the child was white, mixed, black, Indian, Pakistani/Bangladeshi, or belonged to any other ethnic group (Hale & Viner, 2016). *Internalizing* and *externalizing* difficulties were measured at age 11, using the parent‐completed Strengths and Difficulties Questionnaire (SDQ). The emotional and peer subscales of the SDQ were added to create the internalizing difficulties scale, while the conduct and hyperactivity subscales were added to create the externalizing difficulties scale. Both scales ranged from 0 to 20, with higher values suggesting more difficulties (Bozzini et al., 2021; Gutman & Codiroli McMaster, 2020). Finally, *pubertal status* was assessed at age 11 with a binary variable, which showed if the child had yet displayed any signs of puberty or not. Signs of puberty for girls involved body hair, menstruation, and breast growth, while signs of puberty for boys involved body hair, facial hair, and voice change (De Azevedo et al., 2017).

### 2.3 Analytic strategy

**Figure 1:**
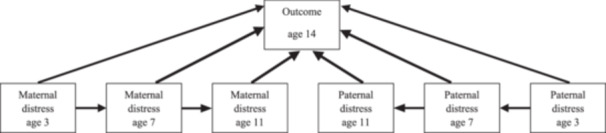
Path analysis diagram.

All analyzes were conducted in Stata (version 15.0). First, to detect any potential sample selection bias, the main study variables and covariates were compared between the analytic (*N* = 11,128) and nonanalytic (*N* = 8115) samples. Next, to gain a better understanding of the data, sex differences were explored for each of the health risk behaviors (girls, *N* = 5619; boys, *N* = 5509). Subsequently, we conducted path analysis, within the framework of generalized structural equation modeling (GSEM), to investigate the paths from paternal and maternal psychological distress to adolescent health risk behaviors. The model used is illustrated in Figure 1. As shown, paternal and maternal psychological distress were modeled simultaneously, and, for both mothers and fathers, earlier distress was allowed to predict later distress (e.g., distress at age 3 was modeled to predict distress at age 7, and distress at age 7 was modeled to predict distress at age 11). Each of the four health risk variables was assessed separately as an outcome. Models were run first unadjusted and then adjusted (such that all age 14 outcomes were regressed on the covariates). The full sample was explored first, and then female and male adolescents separately. To control for the over‐sampling of disadvantaged groups, nonresponse, and attrition, all models were weighted using the *svy* Stata command (Hansen, 2014). The estimator used was observed information matrix (OIM).

Missing data were handled by multiple imputation by chained equations (MICE) (Royston, 2005). All full‐data variables, including outcome variables, were used to predict the missing values. This resulted in 20 imputed datasets, combined for the analysis using Rubin's rules (Royston, 2005). For fathers' psychological distress, missing data was 37.2%, 40.3%, and 37.8%, and for mothers' psychological distress, missing data was 19.4%, 18.5%, and 14.3%, across child ages 3, 7, and 11, respectively.

The analytic sample of our study included households that may not have had a father in all the assessed sweeps or households in which the father figure may have changed. To evaluate the robustness of the results, a sensitivity analysis was performed, including only families with a biological father for our study period, i.e., across child ages 3–14 (sweeps 2–6). This led to a sample of 5156 cases in total, 2583 girls and 2573 boys. Models were run in the same way as in the main analysis, adjusted for covariates.

## 3 RESULTS

### 3.1 Descriptive statistics

Comparisons between the analytic and nonanalytic samples highlight some sample selection bias (Table 1). Specifically, fathers and mothers in the analytic sample had lower levels of psychological distress than those in the nonanalytic sample and were more likely to be university‐educated. Families in the analytic sample were also less likely to be living below the poverty line and more likely to have a biological father living consistently in the household. Mothers in the analytic samples also reported consuming less alcohol, though there were no such difference for fathers. When it comes to offspring variables, it was not possible to compare the prevalence of health risk behaviors, given missing data in the nonanalytic sample leading to small Ns. Nevertheless, adolescents in the analytic sample were more likely to be female and to have presented signs of puberty. Furthermore, they had significantly fewer internalizing and externalizing difficulties. There were no ethnic differences. Supporting Information S1: Table S1 shows the sex differences in the prevalence of each of the outcomes in the analytic sample. Boys were significantly more likely to have drunk alcohol than girls. No other differences emerged.

**Table 1:** Descriptives of the analytic and nonanalytic samples (unweighted data).

		Analytic sample (*N* = 11,128)		Nonanalytic sample (*N* = 8115)		
Categorical variables		*N*	%	*N*	%	*χ* ^2^
Smoking		2351	28.97	557	6.86	1.6e + 03***
Alcohol use		3870	34.78	14	0.17	NA
Binge drinking		1054	9.47	7	0.09	NA
Sexual activity		330	2.97	1	0.01	NA
Girl		5619	50.5	3728	45.9	38.97***
Mixed		322	2.89	196	2.41	3.16
Indian		259	2.33	180	2.22	0.07
Pakistani or Bangladeshi		726	6.52	478	5.89	1.98
Black		385	3.46	278	3.42	0.03
Other		160	1.44	117	1.44	0.04
Father is university‐educated		3272	29.4	76	0.94	21.24***
Mother is university‐educated		4896	44	155	1.91	49.68***
Biological father		5156	46.3	146	1.80	4.8e + 03***
Below the poverty line		3047	27.38	2039	25.13	128.53***
Signs of puberty		6232	56.00	1483	18.27	16.89***
**Continuous variables**	**Range**	***N***	**Mean (SD)**	***N***	**Mean (SD)**	***t***
Paternal distress age 3	[0, 24]	6992	2.86 (3.05)	2916	2.91 (3.34)	0.84
Maternal distress age 3	[0, 24]	8965	3.17 (3.62)	4538	3.51 (4.02)	4.95***
Paternal distress age 7	[0, 24]	6646	2.92 (3.31)	1668	3.11 (3.76)	2.0446*
Maternal distress age 7	[0, 24]	9069	3.01 (3.76)	2822	3.37 (4.03)	4.32***
Paternal distress age 11	[0, 24]	6926	3.80 (3.82)	1281	4.14 (4.32)	2.87**
Maternal distress age 11	[0, 24]	9542	3.89 (4.29)	2293	4.77 (4.94)	8.61***
Internalizing difficulties	[0, 20]	10249	3.12 (3.07)	2537	3.74 (3.54)	8.83***
Externalizing difficulties	[0, 20]	10226	4.30 (3.45)	2536	5.38 (4.02)	13.69***
Paternal alcohol use	[0, 5]	7103	1.64 (1.16)	1321	1.68 (1.24)	0.953
Maternal alcohol use	[0, 5]	9809	1.28 (1.06)	2337	1.35 (1.17)	2.81**

* *p* < .05

** *p* < .01

*** *p* < .001.

### 3.2 Path analysis results

Results from the unadjusted models are presented in the supplement, Supporting Information S1: Tables S2–S5. In all models (unadjusted and adjusted), autoregressive paths were significant, as expected. That is, paternal and maternal psychological distress at age 3 predicted paternal and maternal psychological distress at age 7, respectively. Similarly, paternal and maternal psychological distress at age 7 predicted paternal and maternal distress at age 11, respectively. Findings for the adjusted models are shown in Tables 2, 3, 4, 5, corresponding to smoking, alcohol use, binge drinking, and sexual activity, respectively.

**Table 2:** Smoking results for the adjusted models.

	Full sample	Boys	Girls
	*N* = 11,128	*N* = 5509	*N* = 5619
	*B (SE)*	*B (SE)*	*B (SE)*
PD_age11_ → Smoking	0.02 (0.01)*	0.02 (0.01)	0.02 (0.02)
MD_age11 _→ Smoking	−0.002 (0.008)	−0.008 (0.01)	0.002 (0.01)
PD_age7_ → Smoking	0.01 (0.01)	0.01 (0.02)	0.02 (0.02)
MD_age7 _→ Smoking	0.02 (0.009)*	0.03 (0.01)**	0.01 (0.01)
PD_age3_ → Smoking	−0.007 (0.01)	0.01 (0.02)	−0.02 (0.02)
MD_age3_ → Smoking	−0.003 (0.009)	−0.01 (0.01)	0.006 (0.01)
PD_age7_ → PD_age11_	0.67 (0.02)***	0.66 (0.02)***	0.67 (0.02)***
MD_age7_ → MD_age11_	0.66 (0.01)***	0.67 (0.01)***	0.65 (0.02)***
PD_age3 _→ PD_age7_	0.58 (0.01)***	0.59 (0.02)***	0.57 (0.02)***
MD_age3 _→ MD_age7_	0.56 (0.009)***	0.57 (0.01)***	0.55 (0.01)***

Abbreviations: MD, maternal distress; PD, paternal distress.

* *p* < .05

** *p* < .01

*** *p* < .001.

**Table 3:** Alcohol use results for the adjusted models.

	Full sample	Boys	Girls
	*N* = 11,128	*N* = 5509	*N* = 5619
	*B (SE)*	*B (SE)*	*B (SE)*
PD_age11_ → Alcohol use	0.004 (0.009)	0.01 (0.01)	−0.003 (0.01)
MD_age11_ → Alcohol use	0.004 (0.007)	0.0009 (0.01)	0.005 (0.01)
PD_age7 _→ Alcohol use	0.001 (0.01)	−0.002 (0.01)	0.004 (0.02)
MD_age7 _→ Alcohol use	0.01 (0.008)	0.02 (0.01)*	0.004 (0.01)
PD_age3 _→ Alcohol use	0.002 (0.01)	0.003 (0.02)	0.001 (0.01)
MD_age3_ → Alcohol use	−0.02 (0.008)**	−0.02 (0.01)*	−0.02 (0.01)*
PD_age7_ → PD_age11_	0.67 (0.02)***	0.66 (0.02)***	0.67 (0.02)***
MD_age7 _→ MD_age11_	0.66 (0.01)***	0.67 (0.01)***	0.65 (0.02)***
PD_age3_ → PD_age7_	0.58 (0.01)***	0.59 (0.02)***	0.57 (0.02)***
MD_age3_ → MD_age7_	0.56 (0.009)***	0.57 (0.01)***	0.55 (0.01)***

Abbreviations: MD, maternal distress; PD, paternal distress.

* *p* < .05

** *p* < .01

*** *p* < .001.

**Table 4:** Binge drinking results for the adjusted models.

	Full sample	Boys	Girls
	*N* = 11,128	*N* = 5509	*N* = 5619
	*B (SE)*	*B (SE)*	*B (SE)*
PD_age11 _→ Binge drinking	−0.002 (0.01)	0.003 (0.02)	−0.007 (0.02)
MD_age11 _→ Binge drinking	0.00006 (0.01)	−0.02 (0.02)	0.02 (0.02)
PD_age7_ → Binge drinking	0.009 (0.02)	0.002 (0.02)	0.02 (0.02)
MD_age7_ → Binge drinking	0.02 (0.01)	0.04 (0.02)*	0.0006 (0.02)
PD_age3_ → Binge drinking	−0.002 (0.02)	0.002 (0.02)	−0.006 (0.03)
MD_age3_ → Binge drinking	−0.02 (0.01)	−0.02 (0.02)	−0.02 (0.02)
PD_age7_ → PD_age11_	0.67 (0.02)***	0.66 (0.02)***	0.67 (0.02)***
MD_age7_ → MD_age11_	0.66 (0.01)***	0.67 (0.01)***	0.65 (0.02)***
PD_age3_ → PD_age7_	0.58 (0.01)***	0.59 (0.02)***	0.57 (0.02)***
MD_age3_ → MD_age7_	0.56 (0.009)***	0.57 (0.01)***	0.55 (0.01)***

Abbreviations: MD, maternal distress; PD, paternal distress.

* *p* < .05

*** *p* < .001.

**Table 5:** Sexual activity results for the adjusted models.

	Full sample	Boys	Girls
	*N* = 11,128	*N* = 5509	*N* = 5619
	*B (SE)*	*B (SE)*	*B (SE)*
PD_age11_ → Sexual activity	0.02 (0.03)	0.02 (0.03)	0.03 (0.04)
MD_age11_ → Sexual activity	0.008 (0.02)	0.01 (0.03)	0.002 (0.03)
PD_age7_ → Sexual activity	0.008 (0.03)	0.004 (0.04)	0.01 (0.04)
MD_age7_ → Sexual activity	0.03 (0.02)	0.05 (0.03)	0.02 (0.03)
PD_age3 _→ Sexual activity	−0.02 (0.03)	−0.008 (0.04)	−0.04 (0.04)
MD_age3_ → Sexual activity	−0.001 (0.02)	−0.008 (0.03)	0.006 (0.03)
PD_age7_ → PD_age11_	0.67 (0.02)***	0.66 (0.02)***	0.67 (0.02)***
MD_age7_ → MD_age11_	0.66 (0.01)***	0.67 (0.01)***	0.65 (0.02)***
PD_age3 _→ PD_age7_	0.58 (0.01)***	0.59 (0.02)***	0.57 (0.02)***
MD_age3_ → MD_age7_	0.56 (0.009)***	0.57 (0.01)***	0.55 (0.01)***

Abbreviations: MD, maternal distress; PD, paternal distress.

*** *p* < .001.

When examining smoking, in the full sample, paternal psychological distress at age 11 and maternal psychological distress at age 7 predicted a higher risk. In the boys' subsample, only maternal distress at age 7 predicted a higher risk. In the girls' subsample, there were no significant effects either for paternal or maternal psychological distress.

For alcohol use, maternal psychological distress at age 3 was associated with lower risk, in the full sample analysis as well as separately for boys and girls. Moreover, maternal distress at age 7 predicted a higher risk, though only for boys. Similarly, for binge drinking, maternal distress at age 7 predicted a higher risk for boys. Finally, when it comes to sexual activity, no significant effects occurred.

### 3.3 Sensitivity analysis

Findings from the sensitivity analysis are presented in Supporting Information S1: Tables S6–S9. After adjustments, maternal psychological distress at age 11 increased the risk for binge drinking, in the full sample analysis and for girls. Furthermore, paternal psychological distress at age 11 increased the risk of sexual activity, for girls only. No effects were found among boys or for any other outcomes.

## 4 DISCUSSION

This study aimed to explore the role of timing of paternal and maternal psychological distress in adolescent health risk behaviors, in a large, UK, population sample. Paternal psychological distress was measured at child ages 3, 7, and 11 years, and health risk behaviors at age 14. Maternal psychological distress was examined in the same way as paternal psychological distress was and was modeled in parallel. In general, the effects that emerged showed specificity by both parental gender and type of adolescent outcome as well as clear evidence for the impact of parental difficulties later in childhood. For example, paternal distress at age 11 was associated with a higher risk for smoking. Maternal distress at age 7 was also associated with a higher risk for smoking, as well as a higher risk for alcohol use and binge drinking, though only for boys. Conversely, maternal distress at age 3 predicted a lower risk for alcohol use. For engagement in sexual activity, there were no effects.

We conducted a sensitivity analysis, retaining only families with a biological father in the household across child ages 3–14. Our findings from the main analysis were not replicated, indicating that they may have occurred due to sample selection, and should be interpreted with caution. The sensitivity analysis in this subsample of biological two‐parent intact families showed that maternal and paternal psychological distress at age 11 raised girls' risk for binge drinking and sexual activity, respectively.

Overall, in line with some previous research (Essau & de la Torre‐Luque, 2021), we found that paternal psychological distress is positively linked to adolescent riskiness. The same was the case for maternal psychological distress, supporting past results (Flouri & Ioakeimidi, 2018; Wickham et al., 2015). The mechanisms behind these relationships are unclear, and it is also uncertain if they are the same for fathers and mothers, arguably a priority for future research. One speculation is that distressed parents are likely to engage in risky behaviors themselves (Åhlin et al., 2015; Lawrence et al., 2009), and, according to social‐learning theory, their offspring are likely to imitate them (Bandura, 1978). It is also possible that dangerous practices become normalized or that substances become more accessible (Yap et al., 2017). Another potential mechanism is through lack of monitoring. Distressed parents may struggle with their mental health and day‐to‐day functioning, which limits their capacity to monitor their children's whereabouts. Furthermore, it is possible that they face environmental stressors (e.g., poverty or unemployment), which ‘diverts their attention’ from monitoring. Consequently, it may be easier for their children to engage in dangerous behaviors (Botzet et al., 2019; Kelly et al., 2017).

Our study also suggests the importance of timing for some of these parent distress “effects.” Past studies suggest that maternal psychological distress experienced in late childhood is most impactful for adolescent risky engagement (Flouri & Ioakeimidi, 2018). Our findings regarding maternal distress confirm these conclusions and expand them by demonstrating that such patterns hold for paternal distress as well. Wickham et al. (2015) found that maternal distress in middle childhood is most influential. In our study, while paths emerged for maternal distress at age 7, these were not replicated in our sensitivity analysis. We note that in Wickham et al.'s study (2015) mothers experienced the highest level of symptoms in middle childhood, which may have driven these effects. On the contrary, in our study, both paternal and maternal symptoms were at their highest in late childhood (age 11).

We also note an unexpected finding, as maternal distress at age 3 predicted a lower risk for alcohol drinking. We note that this finding was not replicated in the sensitivity analysis, indicating that it might have occurred due to sample selection bias. Alternatively, mothers with high distress levels at age 3 might have continued to experience elevated distress up to child age 11, which could have affected their offspring in a different way. Specifically, continuous maternal distress could mean that children may undertake significant caretaking responsibilities from a young age, which results in them learning to act more maturely than their peers (Dam & Hall, 2016; Herman‐Stahl et al., 2008). Future studies investigating the impact of chronicity of parental distress could test this hypothesis.

In general, however, our study suggests that the risks for poor child outcomes are greater in the presence of maternal distress, compared to paternal, in line with previous evidence that mothers have a more prominent role in child development than fathers (Weitzman et al., 2011). Mothers are usually the main caregivers, meaning that children are more exposed to maternal than paternal behavior (Weitzman et al., 2011). In line with these arguments, our main analysis yielded more effects for mothers than fathers; however, this was not the case in the sensitivity analysis. There we clearly showed evidence for the importance of distress experienced by both fathers and mothers for girls' risky behaviors in adolescence.

Related to this, both the sensitivity and the main analysis suggest the importance of considering carefully the role of sex for both parent and child. Our results for instance imply that different pathways may be in place for boys and girls. In our main analysis, maternal distress at age 11 emerged as a risk factor for girls, but for boys, maternal distress at age 7 was more impactful. Boys are shown to initiate smoking and drinking before girls (Melotti et al., 2011; Okoli et al., 2013), which could explain why maternal distress at a younger age may be more impactful for them.

This study has some important limitations to consider. First, our analytic sample was more privileged than the nonanalytic sample, in terms of socio‐demographic characteristics and the levels of psychological distress experienced by parents. In families who are more disadvantaged, there may be a different pattern of parent distress effects. Second, even when significant, our effect sizes were generally small. Third, our models did not allow for covariances between paternal and maternal distress scores. Fourth, our focus was to investigate whether paternal (and maternal) distress influence the likelihood of engaging in health‐risk behaviors in adolescence. We did not, however, differentiate these behaviors further, for example, based on their frequency or severity (e.g., some adolescents may have smoked only once, while others may have smoked daily). Fifth, some of our covariates (such as child internalizing/externalizing problems, and parent alcohol use) may not be confounders but likely mediators (i.e., on the causal path), as they were assessed at age 11. Finally, we note that the relationships identified are not causal and that further research is needed to understand the mechanisms underlying them.

Despite the above limitations, this study has several important strengths. Using a large population sample and controlling for key covariates, we found that not only maternal but also paternal psychological distress experienced later in childhood can be a risk factor for adolescent health risk behaviors. To our knowledge, this is the first study to assess these associations longitudinally and to specifically investigate timing effects. Moreover, it is the first to explore whether paternal distress predicts adolescent engagement in sexual activity, with findings indicating that a link may exist but only for girls. Our conclusions highlight that late childhood may be a critical period for the impact of paternal psychological distress, in turn suggesting a clear developmental window for effective prevention of health risk behaviors in adolescence.

## CONFLICT OF INTEREST STATEMENT

The authors declare no conflict of interest.

## ETHICS STATEMENT

The Millennium Cohort Study gained ethical approval from the National Health Service (NHS) Multi‐Center Ethics Committees. The present study has received additional ethical approval from the UCL, Institute of Education's Ethics, all in line with the standards of the 1964 Declaration of Helsinki.

## Supporting information

Supporting information.

## Data Availability

The data that support the findings of this study are openly available in UK Data Service at https://ukdataservice.ac.uk/. Data from the UK's Millennium Cohort Study is publicly available.
