# Supplementary material for: Paternal and maternal psychological distress and adolescent health risk behaviors: The role of sensitive periods
Source: J Adolesc. 2024 Jul 28;96(8):1843–52. doi: 10.1002/jad.12385 (PMC11618725; doi:10.1002/jad.12385)
Supplement: Supplementary file 1 — Supporting information. [file JAD-96-1843-s001.docx]

**Supplement**

**Table S1**

|  | Boys (N=5,509) | | Girls (N=5,619) | |  |
| --- | --- | --- | --- | --- | --- |
|  | *N* | *%* | *N* | *%* | *χ*^2^ |
| Smoking | 1,192 | 21.64 | 1,159 | 20.63 | 0.192 |
| Alcohol use | 1,981 | 35.96 | 1,889 | 33.62 | 0.01** |
| Binge drinking | 521 | 9.46 | 533 | 9.49 | 0.959 |
| Sexual activity | 168 | 3.05 | 162 | 2.88 | 0.605 |

*Prevalence of health risk behaviours for boys and girls*

*Notes:* **p*<0.05, ***p*<0.01, ****p*<0.001

|  | Full Sample  N=11,128 | Boys  N=5,509 | Girls  N=5,619 |
| --- | --- | --- | --- |
|  | *B (SE)* | *B (SE)* | *B (SE)* |
| PD_age11_→ Smoking | 0.03 (0.01)** | 0.03 (0.01)* | 0.03 (0.01)* |
| MD_age11_→ Smoking | 0.02 (0.008)* | 0.004 (0.01) | 0.03 (0.01)* |
| PD_age7_→ Smoking | 0.01 (0.01) | 0.006 (0.02) | 0.02 (0.02) |
| MD_age7_→ Smoking | 0.03 (0.009)** | 0.04 (0.01)** | 0.01 (0.01) |
| PD_age3_→ Smoking | -0.007 (0.01) | 0.01 (0.01) | -0.03 (0.02) |
| MD_age3_→ Smoking | 0.005 (0.009) | -0.005 (0.01) | 0.01 (0.01) |
| PD_age7_→ PD_age11_ | 0.67 (0.02)*** | 0.66 (0.02)*** | 0.67 (0.02)*** |
| MD_age7_→ MD_age11_ | 0.66 (0.01)*** | 0.67 (0.01)*** | 0.65 (0.02)*** |
| PD_age3_→ PD_age7_ | 0.58 (0.01)*** | 0.59 (0.02)*** | 0.57 (0.02)*** |
| MD_age3_→ MD_age7_ | 0.56 (0.009)*** | 0.57 (0.01)*** | 0.55 (0.01)*** |

**Table S2**

*Smoking results for the unadjusted models*

*Notes:* PD=Paternal distress, MD=Maternal distress, **p*<0.05, ***p*<0.01, ****p*<0.001

|  | Full Sample  N=11,128 | Boys  N=5,509 | Girls  N=5,619 |
| --- | --- | --- | --- |
|  | *B (SE)* | *B (SE)* | *B (SE)* |
| PD_age11_→Alcohol use | -0.001 (0.009) | 0.003 (0.01) | -0.005 (0.01) |
| MD_age11_→Alcohol use | -0.002 (0.007) | -0.01 (0.009) | 0.008 (0.009) |
| PD_age7_→ Alcohol use | -0.003 (0.01) | -0.008 (0.01) | 0.002 (0.01) |
| MD_age7_→ Alcohol use | 0.01 (0.008) | 0.02 (0.01) | 0.002 (0.01) |
| PD_age3_→ Alcohol use | -0.004 (0.01) | -0.001 (0.01) | -0.007 (0.01) |
| MD_age3_→ Alcohol use | -0.03 (0.008)*** | -0.03 (0.01)** | -0.02 (0.01)* |
| PD_age7_→ PD_age11_ | 0.67 (0.02)*** | 0.66 (0.02)*** | 0.67 (0.02)*** |
| MD_age7_→ MD_age11_ | 0.66 (0.01)*** | 0.67 (0.01)*** | 0.65 (0.02)*** |
| PD_age3_→ PD_age7_ | 0.58 (0.01)*** | 0.59 (0.02)*** | 0.57 (0.02)*** |
| MD_age3_→ MD_age7_ | 0.56 (0.009)*** | 0.57 (0.01)*** | 0.55 (0.01)*** |

**Table S3**

*Alcohol results for the unadjusted models*

*Notes:* PD=Paternal distress, MD=Maternal distress, **p*<0.05, ***p*<0.01, ****p*<0.001

**Table S4**

|  | Full Sample  N=11,128 | Boys  N=5,509 | Girls  N=5,619 |
| --- | --- | --- | --- |
|  | *B (SE)* | *B (SE)* | *B (SE)* |
| PD_age11_→Binge drinking | -0.002 (0.01) | 0.0009 (0.02) | -0.005 (0.02) |
| MD_age11_→ Binge drinking | 0.006 (0.01) | -0.02 (0.02) | 0.03 (0.02)* |
| PD_age7_→ Binge drinking | 0.002 (0.02) | -0.009 (0.02) | 0.01 (0.02) |
| MD_age7_→ Binge drinking | 0.02 (0.01) | 0.04 (0.02)* | -0.0006 (0.02) |
| PD_age3_→ Binge drinking | -0.009 (0.02) | -0.0004 (0.02) | -0.02 (0.03) |
| MD_age3_→ Binge drinking | -0.02 (0.01) | -0.03 (0.02) | -0.02 (0.02) |
| PD_age7_→ PD_age11_ | 0.67 (0.02)*** | 0.66 (0.02)*** | 0.67 (0.02)*** |
| MD_age7_→ MD_age11_ | 0.66 (0.01)*** | 0.67 (0.01)*** | 0.65 (0.02)*** |
| PD_age3_→ PD_age7_ | 0.58 (0.01)*** | 0.59 (0.02)*** | 0.57 (0.02)*** |
| MD_age3_→ MD_age7_ | 0.56 (0.009)*** | 0.57 (0.01)*** | 0.55 (0.01)*** |

*Binge drinking results for the unadjusted models*

*Notes:* PD=Paternal distress, MD=Maternal distress, **p*<0.05, ***p*<0.01, ****p*<0.001

|  | Full Sample  N=11,128 | Boys  N=5,509 | Girls  N=5,619 |
| --- | --- | --- | --- |
|  | *B (SE)* | *B (SE)* | *B (SE)* |
| PD_age11_→Sexual activity | 0.02 (0.02) | 0.01 (0.03) | 0.03 (0.03) |
| MD_age11_→Sexual activity | 0.01 (0.02) | 0.01 (0.02) | 0.02 (0.02) |
| PD_age7_→Sexual activity | 0.005 (0.03) | -0.002 (0.04) | 0.01 (0.04) |
| MD_age7_→Sexual activity | 0.04 (0.02) | 0.05 (0.03) | 0.02 (0.03) |
| PD_age3_→Sexual activity | -0.03 (0.03) | -0.008 (0.04) | -0.04 (0.04) |
| MD_age3_→Sexual activity | 0.0006 (0.02) | -0.005 (0.03) | 0.007 (0.03) |
| PD_age7_→ PD_age11_ | 0.67 (0.02)*** | 0.66 (0.02)*** | 0.67 (0.02)*** |
| MD_age7_→ MD_age11_ | 0.66 (0.01)*** | 0.67 (0.01)*** | 0.65 (0.02)*** |
| PD_age3_→ PD_age7_ | 0.58 (0.01)*** | 0.59 (0.02)*** | 0.57 (0.02)*** |
| MD_age3_→ MD_age7_ | 0.56 (0.009)*** | 0.57 (0.01)*** | 0.55 (0.01)*** |

**Table S5**

*Sexual activity results for the unadjusted models*

*Notes:* PD=Paternal distress, MD=Maternal distress, **p*<0.05, ***p*<0.01, ****p*<0.001

|  | Full Sample  N=5,156 | Boys  N=2,573 | Girls  N=2,583 |
| --- | --- | --- | --- |
|  | *B (SE)* | *B (SE)* | *B (SE)* |
| PD_age11_→ Smoking | 0.02 (0.01) | 0.02 (0.02) | 0.03 (0.02) |
| MD_age11_→ Smoking | 0.01 (0.01) | 0.001 (0.02) | 0.02 (0.02) |
| PD_age7_→ Smoking | 0.02 (0.02) | 0.02 (0.02) | 0.02 (0.02) |
| MD_age7_→ Smoking | 0.01 (0.02) | 0.02 (0.02) | 0.006 (0.02) |
| PD_age3_→ Smoking | -0.02 (0.02) | -0.0008 (0.03) | -0.04 (0.03) |
| MD_age3_→ Smoking | -0.01 (0.02) | -0.03 (0.02) | 0.004 (0.02) |
| PD_age7_→ PD_age11_ | 0.65 (0.01)*** | 0.63 (0.02)*** | 0.67 (0.02)*** |
| MD_age7_→ MD_age11_ | 0.64 (0.01)*** | 0.63 (0.02)*** | 0.64 (0.02)*** |
| PD_age3_→ PD_age7_ | 0.60 (0.02)*** | 0.63 (0.02)*** | 0.57 (0.02)*** |
| MD_age3_→ MD_age7_ | 0.54 (0.01)*** | 0.56 (0.02)*** | 0.53 (0.02)*** |

**Table S6**

*Sensitivity analysis results for smoking (adjusted models)*

*Notes:* PD=Paternal distress, MD=Maternal distress, **p*<0.05, ***p*<0.01, ****p*<0.001

**Table S7**

|  | Full Sample  N=5,156 | Boys  N=2,573 | Girls  N=2,583 | |  |
| --- | --- | --- | --- | --- | --- |
|  | *B (SE)* | *B (SE)* | *B (SE)* | |  |
| PD_age11_→Alcohol use | 0.009 (0.01) | 0.03 (0.02) | | -0.01 (0.02) | |
| MD_age11_→Alcohol use | 0.02 (0.01) | 0.002 (0.02) | | 0.03 (0.02) | |
| PD_age7_→ Alcohol use | -0.002 (0.01) | -0.008 (0.02) | | 0.007 (0.02) | |
| MD_age7_→ Alcohol use | 0.001 (0.01) | 0.03 (0.02) | | -0.02 (0.02) | |
| PD_age3_→ Alcohol use | -0.005 (0.01) | -0.01 (0.02) | | 0.0009 (0.02) | |
| MD_age3_→ Alcohol use | -0.02 (0.01) | -0.02 (0.02) | | -0.02 (0.02) | |
| PD_age7_→ PD_age11_ | 0.65 (0.01)*** | 0.63 (0.02)*** | | 0.67 (0.02)*** | |
| MD_age7_→ MD_age11_ | 0.64 (0.01)*** | 0.63 (0.02)*** | | 0.64 (0.02)*** | |
| PD_age3_→ PD_age7_ | 0.60 (0.02)*** | 0.63 (0.02)*** | | 0.57 (0.02)*** | |
| MD_age3_→ MD_age7_ | 0.54 (0.01)*** | 0.56 (0.02)*** | | 0.53 (0.02)*** | |

*Sensitivity analysis results for alcohol use (adjusted models)*

*Notes:* PD=Paternal distress, MD=Maternal distress, **p*<0.05, ***p*<0.01, ****p*<0.001

|  | Full Sample  N=5,156 | Boys  N=2,573 | Girls  N=2,583 |
| --- | --- | --- | --- |
|  | *B (SE)* | *B (SE)* | *B (SE)* |
| PD_age11_→Binge drinking | 0.0007 (0.02) | -0.02 (0.03) | 0.02 (0.03) |
| MD_age11_→ Binge drinking | 0.04 (0.02)* | 0.01 (0.03) | 0.07 (0.03)* |
| PD_age7_→ Binge drinking | 0.005 (0.02) | 0.02 (0.03) | -0.008 (0.04) |
| MD_age7_→ Binge drinking | -0.03 (0.03) | 0.008 (0.04) | -0.06 (0.04) |
| PD_age3_→ Binge drinking | -0.008 (0.03) | 0.003 (0.04) | -0.02 (0.04) |
| MD_age3_→ Binge drinking | -0.03 (0.02) | -0.05 (0.04) | -0.02 (0.03) |
| PD_age7_→ PD_age11_ | 0.65 (0.01)*** | 0.63 (0.02)*** | 0.67 (0.02)*** |
| MD_age7_→ MD_age11_ | 0.64 (0.01)*** | 0.63 (0.02)*** | 0.64 (0.02)*** |
| PD_age3_→ PD_age7_ | 0.60 (0.02)*** | 0.63 (0.02)*** | 0.57 (0.02)*** |
| MD_age3_→ MD_age7_ | 0.54 (0.01)*** | 0.56 (0.02)*** | 0.53 (0.02)*** |

**Table S8**

*Sensitivity analysis results for binge drinking (adjusted models)*

*Notes:* PD=Paternal distress, MD=Maternal distress, **p*<0.05, ***p*<0.01, ****p*<0.001

|  | Full Sample  N=5,156 | Boys  N=2,573 | Girls  N=2,583 |
| --- | --- | --- | --- |
|  | *B (SE)* | *B (SE)* | *B (SE)* |
| PD_age11_→Sexual activity | 0.04 (0.03) | 0.002 (0.05) | 0.09 (0.05)* |
| MD_age11_→Sexual activity | 0.02 (0.04) | 0.02 (0.05) | 0.02 (0.06) |
| PD_age7_→Sexual activity | 0.02 (0.04) | 0.009 (0.05) | 0.01 (0.06) |
| MD_age7_→Sexual activity | 0.03 (0.04) | 0.06 (0.05) | -0.01 (0.07) |
| PD_age3_→Sexual activity | -0.05 (0.05) | 0.03 (0.06) | -0.15 (0.08) |
| MD_age3_→Sexual activity | 0.003 (0.04) | -0.01 (0.05) | 0.03 (0.06) |
| PD_age7_→ PD_age11_ | 0.65 (0.01)*** | 0.63 (0.02)*** | 0.67 (0.02)*** |
| MD_age7_→ MD_age11_ | 0.64 (0.01)*** | 0.63 (0.02)*** | 0.64 (0.02)*** |
| PD_age3_→ PD_age7_ | 0.60 (0.02)*** | 0.63 (0.02)*** | 0.57 (0.02)*** |
| MD_age3_→ MD_age7_  *Notes:* PD=Paternal distress, MD=Maternal distress, **p*<0.05, ***p*<0.01, ****p*<0.001 | 0.54 (0.01)*** | 0.56 (0.02)*** | 0.53 (0.02)*** |

**Table S9**

*Sensitivity analysis results for sexual activity (adjusted models)*
